# Turning over DNA methylation in the mind

**DOI:** 10.3389/fnins.2015.00252

**Published:** 2015-07-30

**Authors:** Ryan Lister, Eran A. Mukamel

**Affiliations:** ^1^ARC Center of Excellence in Plant Energy Biology, The University of Western AustraliaPerth, WA, Australia; ^2^The Harry Perkins Institute of Medical ResearchPerth, WA, Australia; ^3^Department of Cognitive Science, University of California San DiegoLa Jolla, CA, USA

**Keywords:** DNA methylation, epigenome, brain, memory, learning, demethylation

## Abstract

Cytosine DNA methylation is a stable epigenetic modification with established roles in regulating transcription, imprinting, female X-chromosome inactivation, and silencing of transposons. Dynamic gain or loss of DNA methylation reshapes the genomic landscape of cells during early differentiation, and in post-mitotic mammalian brain cells these changes continue to accumulate throughout the phases of cortical maturation in childhood and adolescence. There is also evidence for dynamic changes in the methylation status of specific genomic loci during the encoding of new memories, and these epigenome dynamics could play a causal role in memory formation. However, the mechanisms that may dynamically regulate DNA methylation in neurons during memory formation and expression, and the function of such epigenomic changes in this context, are unclear. Here we discuss the possible roles of DNA methylation in encoding and retrieval of memory.

## Introduction

A fundamental aim of neuroscience is to understand the molecular, cellular and network mechanisms for encoding, storage and expression, or recall, of memory. Inspired by the prominence of synaptic connections between neurons in the physical architecture of brain circuits, theorists of brain function have long considered synapses to be the locus of information storage and processing. As suggested by Donald Hebb, neural activity could induce changes to the strength of synapses and thereby alter future network activity in an information-preserving manner (Hebb, 1949). Since then, computational neuroscience research on learning and memory has concentrated on the ways in which networks of neurons connected by plastic synapses can give rise to the processes of memory. Meanwhile, molecular and cellular neurobiology continues to elucidate the mechanisms of neural activity-triggered strengthening and weakening of synapses, known as long term potentiation (LTP) and depression (LTD), respectively (Kessels and Malinow, 2009). This synapse-centered framework for memory research is increasingly successful, and it has enabled *in vivo* fluorescence microscopy to visualize the synaptic changes that accompany encoding of new memories (Lamprecht and LeDoux, 2004).

However, despite its successful record, the synaptic theory of learning and memory cannot account for all of the empirical observations. Both transcription of genes and translation of proteins, including translation occurring in ribosomes located in neuronal dendrites near the activated synapses, are necessary for LTP and LTD (McClung and Nestler, 2008). The gene regulatory contribution to activity-dependent plasticity is mediated by multiple pathways, including CREB/MAPK (Cortés-Mendoza et al., 2013). These findings raise the question of whether gene regulation plays a merely permissive role for memory storage, for example, by synthesizing the ion channels, receptors, trafficking proteins and other cellular components necessary for altering synapse strength? Or, alternatively, does gene regulation play an instructive role in synaptic plasticity and memory formation, enabling sophisticated and information-rich responses to specific activity patterns, which can be stably maintained or dynamically modulated? If so, mechanisms of gene regulation could influence cellular and synaptic physiology in a complex way that meaningfully contributes to the brain's computational function. Indeed, every mammalian cell possesses a sophisticated and highly specialized network of epigenetic mechanisms that control gene expression over a broad range of timescales. An intriguing possibility is that cells, and in particular post-mitotic neurons, take advantage of such epigenetic information processing to support cognitive processes (Crick, 1984; Day and Sweatt, 2011). A key implication would be that genomic and epigenomic regulation should be considered as central elements, and not merely implementational details, in computational models of biological cognition.

Recently, this more expansive hypothesis for the role of epigenetic gene regulation in memory formation has been bolstered by evidence that covalent modifications of DNA and chromatin participate in neuronal adaptation to experience. In this review, we explore the hypothesis that DNA methylation, one of the best characterized epigenetic regulatory mechanisms, could play an instructive role in memory encoding and storage. We describe the landscape of DNA methylation in brain cells, including unique features of the neuronal methylome that suggest neurons may use distinct modes of epigenetic regulation that are not present in other cell types. In particular, we discuss evidence that DNA methylation is dynamically regulated in brain cells, with enzymatically controlled deposition and removal of methylation marks in response to neural activity. We conclude with a perspective on the potential implications of dynamical DNA methylation for the processes of memory, and future directions that will be crucial for further exploration of this possibility.

## Unique features of the brain methylome

DNA methylation patterns are highly dynamic through mammalian development, with numerous cell-type specific methylation patterns detected between distinct differentiated cell types (Maegawa et al., 2010; Maunakea et al., 2010). DNA methylation patterns are established by the *de novo* DNA methyltransferases DNMT3A and DNMT3B, while DNMT1 maintains DNA methylation patterns following genome replication (Yoder et al., 1997; Bestor, 2000; Goll and Bestor, 2005). Furthermore, the catalytically inactive DNMT3L protein interacts with its paralogs DNMT3A and DNMT3B, acting as an adaptor protein that can stimulate the DNA methyltransferase activity (Chédin et al., 2002; Gowher et al., 2005; Wienholz et al., 2010). DNMT3L plays important roles in establishing DNA methylation patterns in gametogenesis and in embryonic stem cells (Neri et al., 2013; Vlachogiannis et al., 2015). Both DNMT1 and DNMT3a have been shown to maintain DNA methylation and regulate synaptic function in adult forebrain neurons (Feng et al., 2010). DNA methylation in the genome of most vertebrate tissues is almost exclusively located at CG dinucleotides (also called CpG sites), and has most commonly been studied in this context. The advent of high-throughput DNA sequencers has enabled deep sequencing of sodium bisulfite-converted genomic DNA, allowing identification of the exact sites, sequence context, and levels of DNA methylation throughout almost entire eukaryotic genomes, termed the DNA “methylome.” This approach has shown that the methylome of brain cells has several unique features compared with other mammalian cell types. First, DNA methylation in the CH context (mCH, where H = A, C, or T) has been identified in the brain in both neurons and glial cells (Ramsahoye et al., 2000; Xie et al., 2012; Lister et al., 2013; Guo et al., 2014). This atypical feature of the brain methylome is also present in embryonic stem cells, but is much less abundant in other differentiated tissues (Lister et al., 2009; Schultz et al., 2015). Second, there is a substantial enrichment of 5-hydroxymethylcytosine (5hmC) in brain cells (Kriaucionis and Heintz, 2009). Below we discuss in detail these different forms of DNA methylation identified in the mammalian brain.

### Non-CG methylation

**Figure 1:**
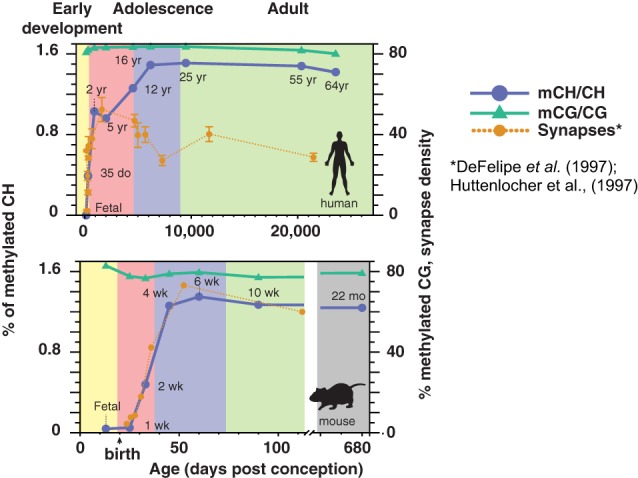
**Developmental dynamics of DNA methylation in the human and mouse brain**. During early post-natal development in humans and mice, mCH rapidly accumulates in the brain in parallel with synaptogenesis. Synaptic density is shown as synapses per 100 μm^2^ (mouse) or per 100 μm^3^ (human). (DNA methylation figure is adapted from Lister et al., 2013; synaptic density data are adapted from Huttenlocher and Dabholkar, 1997 and Morris et al., 2014).

While DNA methylation is present in the conventional CG context in neurons and glia from before birth, mCH is almost undetectable in fetal and early-infant brain cells. Starting around 1 week of age in mice and within the first 2 years in humans (Figure 1), mCH accumulates rapidly and in parallel with synaptogenesis and synaptic pruning as the brain matures (Lister et al., 2013). By adulthood, the abundance of mCH has grown to a level equivalent to mCG in the neuronal genome, and in humans mCH accounts for more than half of all neuronal methylcytosines. This mCH appears to require expression of Dnmt3a, as shown by a recent conditional knockout in mouse neurons that eliminated mCH in the cerebellum (Gabel et al., 2015). Both mCH and mCG are strongly anti-correlated with gene expression in both neurons and glia, suggesting that mCH might play a previously unrecognized role in the repression of gene expression in neurons. Indeed, mCH was reported to repress transcription in reporter assays in mouse neurons, while a conditional neuronal triple knockout of all three *DNMTs* led to reduced neuronal mCH but had little effect upon mCG (Guo et al., 2014). In support of this, glial genomes, which have only 10–20% as much methylation in the CH context compared to neurons, show highly localized mCH hypermethylation within gene bodies of repressed genes that are specifically active within neurons. These genes are specifically depleted of mCH (hypomethylated) in neurons, showing a cell type-specific role for mCH.

In addition to its broad genomic distribution, mCH is widely distributed across brain regions, mammalian species, and in multiple neuronal cell types. Besides the human and mouse frontal cortex (Xie et al., 2012; Lister et al., 2013), abundant mCH has been observed in the mouse dentate gyrus (Guo et al., 2014), in chimpanzee prefrontal cortex (Zeng et al., 2012), and mouse cerebellum (Gabel et al., 2015). Importantly, mCH in neurons and glia is most abundant at CAC positions, which is distinct from the preferred sequence context in embryonic stem cells and pluripotent cell lines (Ziller et al., 2011; Varley et al., 2013). A key question concerns whether mCH is differentially distributed across brain cell types. Cortical function relies on a balance of activity among diverse neuron types, including excitatory pyramidal cells and a wide variety of inhibitory interneurons. These neural populations arise from distinct progenitor pools located in separate brain regions, and they follow different developmental trajectories. Neurons signal via a variety of neurotransmitters and they differ in terms of multiple morphological and physiological characteristics that affect their role in supporting healthy brain network dynamics. Epigenetic profiling of specific brain cell types in adults remains technically challenging (Maze et al., 2014), but recent advances will allow profiling mCG and mCH with greater cell type specificity (Smallwood et al., 2014; Farlik et al., 2015; Mo et al., 2015). mCH, alongside mCG and other epigenetic mechanisms, thus represents a potentially information-rich substrate for shaping the cell type-specific epigenetic landscape of neurons.

### Hydroxymethylcytosine

A second unique feature of the brain methylome is the presence of 5-hydroxymethylcytosine (5hmC). Together, mC and 5hmC can be considered as the fifth and sixth bases that, alongside A, G, T, and unmodified C, make up an epigenetically enhanced DNA code. 5hmC is highly enriched in brain tissue and is particularly concentrated in specific neuron types, such as cerebellar Purkinje cells where it is estimated to be around 40% as abundant as mCG (Penn et al., 1972; Kriaucionis and Heintz, 2009; Mellén et al., 2012). 5hmC accumulates in multiple brain areas during development (Szulwach et al., 2011), not unlike the developmental accumulation of mCH. Interest in 5hmC was stimulated by the identification of specific pathways for converting mC to 5hmC via the TET family enzymes (Tahiliani et al., 2009). Further oxidation coupled with mechanisms such as the base-excision repair pathway can lead to demethylation of sites marked by 5hmC (He et al., 2011; Wu et al., 2014). These findings have raised the possibility that the abundant 5hmC found in mammalian neurons is a transient, intermediate state at locations undergoing active demethylation. According to this view, some genomic locations may undergo a cyclical dynamic of methylation and demethylation, leading to a steady-state distribution of 5hmC in a subset of cells at any point in time. Alternatively, 5hmC in neurons could represent a stable mark that is not a precursor to further modification or demethylation (Hahn et al., 2013). Instead, developmental accumulation of 5hmC in the brain may be associated with the loss of chromatin marks associated with polycomb-mediated repression, such as H3K27me3 (Hahn et al., 2013). Further experiments to distinguish these possibilities might manipulate specific elements of the proposed methylation/demethylation pathways, preferably with both temporal and cell type specificity (Wu et al., 2014). 5hmC is enriched at specific functional genomic compartments, including actively transcribed gene bodies (Mellén et al., 2012; Hahn et al., 2013; Lister et al., 2013). It can be recognized by the transcription factor MeCP2, suggesting that it could play a role in cognitive function (Mellén et al., 2012). Intriguingly, mCH may also be capable of binding MeCP2 and inducing transcriptional repression (Chen et al., 2015).

The above studies established that, in addition to classical methylation at CG positions, mammalian neurons accumulate two forms of DNA methylation that are unusual outside of the brain and pluripotent cells: mCH and 5hmC. What is the relationship between these two neuronally-enriched epigenetic marks? Bisulfite sequencing cannot, by itself, distinguish mC and 5hmC. Using TET-assisted bisulfite sequencing (TAB-Seq) 5hmC was found to be restricted to the CG context in mouse and human embryonic stem cells (Yu et al., 2012) and in mouse fetal and adult frontal cortex (Lister et al., 2013). mCH and 5hmC thus act as independent epigenetic regulatory marks, affecting distinct genomic sites.

## The multi-scale brain methylome

**Figure 2:**
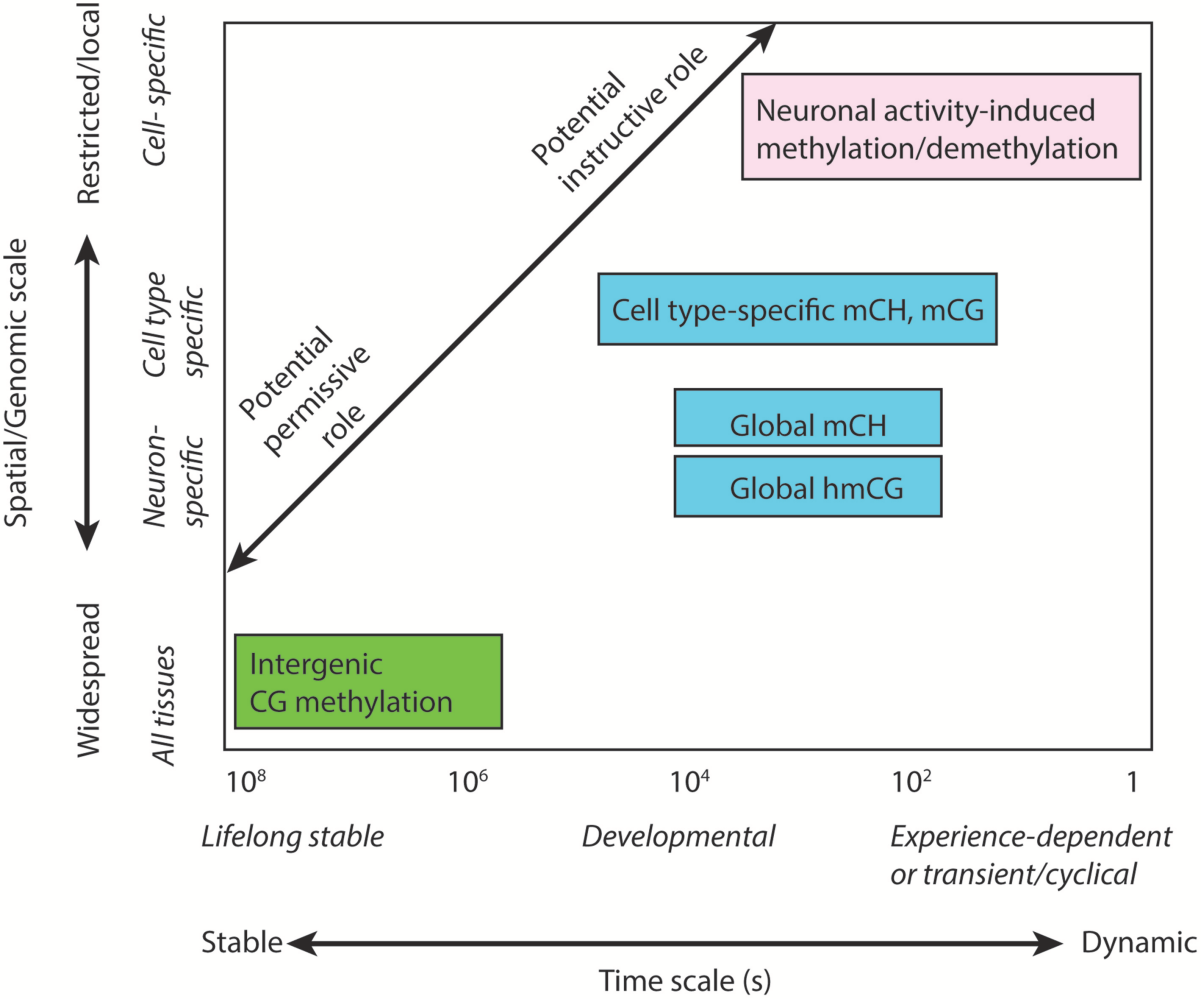
**Multi-scale analysis of DNA methylation in the brain**. Distinct sub-types of DNA methylation form features that exist on different spatial scales within the genome and organism, and have the potential to operate or persist over a wide range of time scales. DNA methylation features with different spatio-temporal characteristics may facilitate distinct permissive or instructive roles in brain function, memory and learning.

To understand the potential role of different DNA modifications in cognition, it is helpful to classify them according to their temporal, spatial and genomic scale (Table 1; Figure 2). The most widespread, stable methylation patterns, such as extensive CG methylation outside of CG islands and distal regulatory elements, are shared across cell types and brain regions, persist through cellular differentiation and brain development, and are not generally altered as a function of experience. Such marks may be necessary for cellular function, as evidenced by the lethality of disruption of the maintenance methyltransferase Dnmt1 (Liao et al., 2015). However, constitutive methylation is not suited to a role in information processing, which requires a flexible, high-entropy substrate for encoding and storing the traces of specific experiences.

**Table 1:** **The multi-scale DNA methylome**.

**Process type**	**Genomic scale**	**Cellular scale**	**Spatial scale**	**Time scale**	**Examples**	**Potential permissive/instructive for learning and memory**	**References**
Constitutive	Genome-wide	All adult cells	Brain-wide	Weeks/Years (lifespan)	Repressive role of promoter mCG	Permissive	
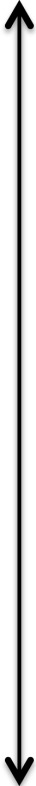							
	Genome-wide	Neurons	Brain-wide	Weeks/Years (lifespan)	Accumulation of mCH	Permissive? Or no role?	Xie et al., 2012; Lister et al., 2013
					Accumulation of hmCG		Kriaucionis and Heintz, 2009; Szulwach et al., 2011; Mellén et al., 2012
	Genome-wide			Dynamic (hours?)	Reduction of global mC following neuronal depolarization or fear conditioning	Likely permissive	Ma et al., 2009; Guo et al., 2011a
	Chromosomal	All adult cells	Brain-wide	Weeks/Years (lifespan)	X-inactivation	Permissive	Lee and Bartolomei, 2013
	Megabase	All adult cells	Brain-wide	Weeks/Years (lifespan)	Differentially methylated valleys (DMVs); mCH deserts	Permissive	Xie et al., 2013
	500 bp	Specific neuron or glial cell types	Brain-wide	Weeks/Years (lifespan)	Cell type DMRs	Permissive + Instructive?	Lister et al., 2013; Ziller et al., 2013
	500 bp	Specific neuron or glial cell types	Local brain region	Weeks/Years (lifespan)	Cell type and regional DMRs	Permissive + Instructive?	
	500 bp	Individual cells	Local	Dynamic (hours?)	Activity-dependent DMRs	Potentially Instructive?	Lubin et al., 2008; Miller et al., 2010; Guo et al., 2011a,b; Mizuno et al., 2012; Baker-Andresen et al., 2013; Day et al., 2013
					Dynamic methylation at Reln, CaN, Egr1		Miller et al., 2010
	500 bp?	Individual cells		Dynamic (hours?)	Activity-dependent 5hmC (DhMRs)		Hahn et al., 2013; Li et al., 2014
Local	10 bp	Specific neuron or glial cell types		Weeks/Years (lifespan)	Demethylation at (activity-dependent or independent) transcription factor binding sites	Potentially instructive?	Guo et al., 2011b

*Examples of DNA methylation features that exist at different spatio-temporal scales*.

More dynamic aspects of the neuron methylome include the accumulation of abundant mCH and 5hmC in neurons during brain development (Kinde et al., 2015). These processes unfolds over weeks (in mice) or years (human). These forms of DNA methylation affect the entire genome and, in the case of mCH, multiple neuron and glial cell types. These global patterns are thus more likely to play a neuron-specific role and may contribute to synaptic plasticity (see below). Yet, their widespread distribution makes them unlikely candidates for an information processing function.

Finally, discrete genomic regions, including gene bodies, promoters, and distal enhancers, show cell type- and brain region-specific mCG and mCH patterns (Lister et al., 2013; Ziller et al., 2013). In principle, some of these locations could be modulated in specific assemblies or circuits of cells; for example, a group of cells that contribute to a particular remembered place representation in the CA1 region of the hippocampus could, in response to a shared pattern of synaptic input, experience a coordinated modification of their DNA methylation state at particular genes or regulatory elements. Current DNA methylome profiling techniques lack the sensitivity or resolution to identify such cell-specific methylation patterns (Maze et al., 2014), which may require bisulfite sequencing of material from small samples or even individual neurons (Smallwood et al., 2014; Farlik et al., 2015). If such patterns do exist and are at least partially regulated rather than stochastically modulated, they would be candidates for an instructive role in memory storage and information processing in the brain. They could potentially affect cellular behavior in specific and adaptive ways. However, linking these forms of DNA methylation to cognition requires understanding (1) how perturbing specific elements of the methylome may affect memory; and (2) how experience and neuronal activity can influence, and in turn be influenced by, discrete changes to individual cells' methylome. Next, we turn to emerging evidence linking memory formation and storage with dynamic methylation patterns in brain cells.

## DNA methylation is needed for neuronal plasticity and memory

Central to the processes of learning and memory is neuronal plasticity, the ability of neuronal activity to trigger lasting changes in the number and strength of synaptic connections between neurons (De Roo et al., 2008). In addition to such Hebbian plasticity, modulation of cellular properties such as intrinsic excitability or synaptic scaling can also contribute to neural plasticity (Guzman-Karlsson et al., 2014). To enable healthy neural plasticity, post-mitotic neurons of the adult brain must establish and maintain specific states of gene expression following neuronal activity in order to sustain long-term synaptic responses. Epigenetic regulatory pathways could play a key role by imparting stable states of transcriptional activity. It is now clear that epigenetic processes play critical roles in activity-dependent regulation of gene expression, and are required for adult neurogenesis, synaptic plasticity, and memory formation, consolidation and extinction (Lim et al., 2006; Miller and Sweatt, 2007; Schor et al., 2009; Feng et al., 2010; Gräff et al., 2012; Cortés-Mendoza et al., 2013; Day et al., 2013). *Dnmt1* and *Dnmt3a* mRNA, protein, and activity are reduced by neuronal membrane depolarization (Sharma et al., 2008), and contextual fear conditioning increases *Dnmt3a* and *Dnmt3b* expression in the hippocampus (Miller and Sweatt, 2007). These data suggest that neural activity may modulate the abundance and activity of the cellular DNA methylation machinery.

### Genetic manipulation of DNA methyltransferases

Disruption of the cellular pathways that establish, modify and maintain DNA methylation patterns in rodents has revealed that DNA methylation is required for learning and memory (Miller and Sweatt, 2007; Feng et al., 2010; Day et al., 2013). Conditional ablation of *Dnmt1* impaired the postnatal viability, maturation and function of CNS neurons, causing abnormal excitability and dendritic arborization, impaired synaptic long-term potentiation, and learning and memory deficits (Golshani et al., 2005; Hutnick et al., 2009). Conditional knockout of *Dnmt3a* in mouse forebrain neurons has implicated the *de novo* methyltransferase in specific complex cognitive and synaptic processes. While no differences in basal synaptic transmission were observed, *Dnmt3a* cKO mice displayed impaired memory formation and abnormal fear extinction, as well as spatial object memory, and induction and maintenance of hippocampal LTP (Feng et al., 2010; Morris et al., 2014). Thus, the DNA methyltransferases expressed in the adult brain appear to be essential for complex neuronal functionality, cognition, and adult behavior.

Consistent with this proposed role, the expression of *Dnmt3a* declines in the mouse hippocampus and cortex with increasing age in parallel with decreases in cognitive performance, hippocampus dependent memory, and euchromatic DNA methylation levels (Kang et al., 2001; Oliveira et al., 2012). A specific isoform of this methyltransferase, *Dnmt3a2*, is transcribed in response to neuronal activity, similar to other immediate early genes, and learning-induced *Dnmt3a2* induction was reduced in the brains of aged mice (Oliveira et al., 2012). Strikingly, adenoviral delivery and expression of *Dnmt3a2* in the hippocampus caused an increase in global DNA methylation levels in infected neurons and improved memory performance in fear conditioning and spatial object recognition tests. Conversely, shRNA-mediated knockdown of *Dnmt3a2* in mature hippocampal neurons impaired long-term memory formation (Oliveira et al., 2012). Together, these findings suggest that activity-induced modulation of DNA methylation machinery and patterns in mature neurons is functionally important in memory formation, and that progressive reduction in *Dnmt3a* is involved in age-related cognitive decline in the mammalian brain.

Genetic manipulations also suggest a cognitive role for the demethylation machinery. Several recent studies have reported that impairment of the Tet dioxygenase enzymes results in impaired learning and memory. Tet1 knockout mice displayed impaired fear memory extinction and aberrantly stronger hippocampal long-term depression, together with reduced neural 5hmC and decreases in expression of neuronal activity-regulated genes that were associated with increased promoter DNA methylation (Rudenko et al., 2013). Conversely, viral-mediated overexpression of Tet1 led to a global increase in 5hmC and a decrease in mC, and impaired formation of long-term memory in a contextual fear conditioning paradigm (Kaas et al., 2013).

### Pharmacological inhibition of DNA methyltransferases

In addition to genetic disruption of DNA methylation pathways, pharmacological inhibition of DNA methyltransferase activity has been widely used to investigate the role of this modification in neurological function. Incorporation of nucleoside analogs such as 5-aza-2′-deoxycytidine (5-azacytidine) and zebularine into genomic DNA results in hypomethylation due to their formation of a covalent bond with DNMTs, while RG108 acts as a non-nucleoside direct inhibitor of DNMTs through binding to the active site of the enzymes. Administration of these inhibitors has been used to study the involvement of DNA methylation in memory formation. For example, LTP can be blocked by infusion of DNMT inhibitors into hippocampal tissue slices, resulting in rapid demethylation of the promoters of *reelin* and *Bdnf*, which encode factors involved in synaptic plasticity (Levenson et al., 2006). Furthermore, contextual fear conditioning in rodents was reported to increase *Dnmt* expression in the hippocampus, and methylation and silencing of *protein phosphatase 1*, which encodes a chromatin remodeling regulator involved in memory suppression. *Dnmt* inhibition in this paradigm impeded memory formation (Genoux et al., 2002; Miller and Sweatt, 2007; Koshibu et al., 2009) and LTP (Miller et al., 2008). Moreover, infusion of DNMT inhibitors into the prefrontal cortex reduced the induction of promoter methylation at the memory suppressor gene *calcineurin* (*CaN*). This resulted in reduction of remote fear memory when administered and tested 1 month after training, but had no effect only 1 day after training, consistent with a role for DNA methylation in mediating long-term memory (Miller et al., 2010). Notably, 5-azacytidine, zebularine and RG108 were all demonstrated to disrupt remote memory. In a separate study, through infusion of 5-azacytidine into different brain regions in a cocaine-induced learning and memory model in mice, it was found that DNA methylation is required in the hippocampus for learning, and in the prelimbic cortex for memory retrieval (Han et al., 2010). Moreover, Day et al. reported that reward-related memory formation was associated with changes in DNA methylation in immediate early genes in the ventral tegmental area (VTA); the learning could be blocked through RG108 administration in the VTA (Day et al., 2013). The effect of DNA methylation on reward learning was remarkably specific to a localized brain region: disruption of methylation in brain regions adjacent to the VTA had no effect on learning (Day et al., 2013).

While the use of DNMT inhibitors has indicated a wide range of potential roles for DNA methylation in neuronal plasticity and memory, it remains unknown how DNMT inhibition through administration of nucleoside analogs such as 5-azacytidine and zebularine result in demethylation in post-mitotic neurons. This effect is puzzling given that the inhibitory effect of the drugs is understood to depend on incorporation into genomic DNA during genome replication. One potential mode of action could be through inhibition of cyclical demethylation-methylation processes, for example by nucleoside analog incorporation into genomic DNA after Tet- and base excision repair-mediated demethylation of 5hmC, followed by inhibition of DNMT-mediated remethylation of the cytosine analog.

These studies provide experimental support for a potential role of DNA methylation and demethylation in memory formation and synaptic and systems memory consolidation. Together, they suggest that the DNA methylation machinery and modification states in the brain may respond to experience, and could play important roles in neuronal plasticity and memory formation. However, it should be noted that the observed changes in DNA methylation in such studies are often assessed only at candidate loci, mostly at CG islands and promoter regions, consider methylation only in the CG context, and often report only partial changes in the level of methylation in the surveyed cell population. In order to gain insights into the full extent of the DNA methylation dynamics in the brain related to neuronal plasticity and memory, it will be important to comprehensively assess all forms of DNA methylation, including mCH and 5hmC. Such studies will ideally generate single base resolution profiles throughout the entire genome, in specific subsets of cells, or even at the single cell level. Continuing advances in single cell DNA methylome analysis (Smallwood et al., 2014; Farlik et al., 2015) and techniques for *in vivo* neuronal monitoring and manipulation (Deisseroth and Schnitzer, 2013), combined with decreases in the cost of DNA sequencing, will be critical for future progress in this area.

## Evidence for dynamic methylation in brain cells

The emergence of a potential role for DNA methylation in the dynamic regulation of neural circuits is surprising, given the remarkable stability and conservation of the methylome. A survey of DNA methylation patterns in a range of tissues and cell lines found evidence for dynamic methylation at only ~22% of CG positions (Ziller et al., 2013). Whole-genome profiling studies have found highly consistent levels of methylation at individual CG and CH positions between frontal cortex samples in different adult mice or human subjects (Pearson *r*>0.8, nearly as high as could possibly be observed given the sampling statistics; Lister et al., 2013). Homologous CH positions in human and mouse frontal cortex also showed highly conserved methylation (Lister et al., 2013). A large-scale study of DNA methylation in frontal cortex of 738 aged human subjects found that levels of mCG were significantly, but very weakly, correlated with sex, age, Alzheimer's disease status, and, intriguingly, the time of the subject's death (Lim et al., 2014). If different adult individuals, with different life experiences, have precisely conserved patterns of mCG and mCH in their frontal cortex, then how much room is there for neuronal activity-dependent modulation? Yet, even within a largely conserved methylome that is highly consistent between different brain cells and across individuals and species, dynamic modulation of methylation at specific sites or regions could have an important impact. Localized dynamic methylation within key gene regulatory regions would not contradict the overall conservation of methylation at a global level.

Consistent with this, a growing number of studies have shown that DNA methylation can be dynamically modified by neuronal activity or by memory-forming experiences. However, the extent of such changes remains largely unknown due to the limited capability of current methylome profiling to provide cell type-specific, genome-wide and base-resolution information. At a global level, the amount of both mC and 5hmC can be significantly reduced by neuronal activity, such as seizures induced in the hippocampus (Kaas et al., 2013). By using methyl-sensitive cut counting (MSCC), a restriction-enzyme based approach that profiles ~220,000 CG sites (around 1% of all CG positions) distributed throughout the genome, Guo et al. showed that ~1.4% of the sampled CG sites may gain or lose methylation in brain cells following neuronal depolarization or exercise (Guo et al., 2011a). Interestingly, sites of dynamic methylation were enriched at intergenic regions with low CG density (Guo et al., 2011a), suggesting the activity-induced changes in methylation may preferentially occur at distal regulatory elements.

Neuronal activity has been reported to modulate the activity of the mC machinery (Miller and Sweatt, 2007; Sharma et al., 2008; Guo et al., 2011a) resulting in altered patterns and levels of DNA methylation and thus playing critical roles in learning and memory formation (Miller and Sweatt, 2007; Day et al., 2013). Similarly, neural activity and memory-inducing experiences can modulate expression of enzymes involved in conversion of mC to 5hmC, including Tet1 (Kaas et al., 2013) and Tet3 (Li et al., 2014; Yu et al., 2015). Knockout or over-expression of Tet1 impacted expression of multiple immediate early genes, in addition to the alterations in DNA methylation patterns and contextual memory noted above (Kaas et al., 2013; Rudenko et al., 2013). Moreover, as mature neurons do not divide, any changes in neuronal DNA methylation have the potential to be stable since they will not be passively lost during genome replication. Such stability is especially important for mCH, which has no known mechanism for faithful duplication of the methylation pattern during cell division as occurs for mCG via Dnmt1 activity. DNA methylation could therefore provide a means by which patterns of transcriptional activity could be modified and stably maintained over long periods of time. Thus, these recent findings suggest that neuronal activity-induced modulation of DNA methylation may be a molecular mechanism of memory storage that plays critical roles in synaptic plasticity, learning and memory in the adult brain. These alterations in the activity of methylation enzymes and in global levels of methylation could be consistent with a permissive role in learning and memory, but they do not directly indicate a more sophisticated, instructive role.

To explore such a role, some studies have focused on DNA methylation within specific genomic locations. Often, these loci are selected based on prior knowledge or assumptions regarding the molecular pathways involved in particular forms of learning. These locations are often CG-rich promoters or exons of genes with known neuronal activity-dependent regulation, such as *Bdnf* (Martinowich et al., 2003; Lubin et al., 2008), *Reelin, Calcineurin*, and *Egr1* (Miller et al., 2010). Some genes were found to be persistently demethylated at CG sites following fear conditioning (*Egr1*), while others gained mC during a transient window lasting several hours after training (*Reelin*). Other genes were affected with a late time course: methylation of the promoter of the memory suppressor *calcineurin* (*CaN*) in the rat prefrontal cortex occurred within 1 day of training, but not after 1 h, and persisted for at least 30 days (Miller et al., 2010). In addition to contextual fear conditioning, early life experiences including maternal care can have lifelong behavioral consequences that are linked with dynamic DNA methylation at sites such as the glucocorticoid receptor *Nr3c1* and *Gad1* promoter (Weaver et al., 2004).

Dynamic hydroxymethylation (5hmC) has also been reported in multiple forms of learning. Gephyrin is directly involved in the extinction phase of fear conditioning, and it was found to harbor increased 5hmC and decreased mC that correlated with increased expression 24 h post-extinction (Li et al., 2014). Reward learning and addiction pathways are also linked with dynamic DNA hydroxymethylation (Feng and Nestler, 2013); levels of Tet1 expression were reduced, while 5hmC was altered at ~10,000 genomic locations in the nucleus accumbens of mice exposed to cocaine (Feng et al., 2015). Reward learning experiments showed that experience-dependent dynamic methylation is targeted to specific genes (Day et al., 2013). Manipulation of Tet1 levels in the mouse brain has also been reported to modify DNA methylation at loci that display activity-induced DNA demethylation. AAV-mediated overexpression of Tet1 was shown to result in demethylation of the promoters of *Bdnf* and *Fgf1B*, which were previously observed to undergo active demethylation in response to electroconvulsive stimulation (ECS) (Martinowich et al., 2003), while shRNA-mediated knockdown of *Tet1* through AAV delivery in the mouse brain abrogated ECS-induced *Bdnf* and *Fgf1B* promoter demethylation (Ma et al., 2009; Guo et al., 2011b).

A critical experimental and conceptual challenge is integrating results about dynamic DNA methylation from genome-scale profiling with findings at the level of single genes, promoters or enhancers. How widespread are changes in DNA methylation during learning? The genetic, molecular and cellular interactions that mediate different forms of learning and memory are intricate, and likely include many redundant and complementary pathways that could affect multiple genes and distal regulatory regions. Importantly, studies that reported learning-related changes to DNA methylation in activity-regulated neuronal genes like *Egr1* also found no change at housekeeping genes like *Gapdh* (Day et al., 2013). Yet, such selective sampling does not address the vast majority of the potential learning-related methylome dynamics. It is therefore essential to analyze genome-wide data that interrogate the dynamic methylome without prior biases.

However, using genome-scale profiling techniques such as MethylC-seq or TAB-seq (discussed above) it may be difficult to achieve the sensitivity required for detecting DNA methylation changes of ~5-10% that are frequently reported by studies using targeted sequencing, and which reflect changes occurring in only a small sub-population of cells. This limitation arises because the expense of genome-wide profiling is often prohibitive for generating high coverage (say, >30-fold) for multiple replicates (Shin et al., 2014). Even if such data were available, a conceptual challenge remains in interpreting the many changes in mC and 5hmC that correlate with specific experiences or forms of neural activity. Some of these methylation dynamics may play a causal role for downstream modulation of transcriptional activity, for example by altering the binding of methylation-sensitive transcription factors (Schübeler, 2015), while others may be a consequence rather than a driver of changes in transcription. Such dynamic methylation could have different effects in different cell types, so cell type-specific profiling is an essential goal.

## Outlook and open questions

Although the presence of methylated nucleotides in DNA has long been known, the development of tools for high-throughput, genome-wide profiling (Maze et al., 2014) has established that the landscape of DNA methylation is complex and highly regulated at multiple spatial and temporal scales. In mammalian neurons, the methylome is distinguished from other cell types by the abundant presence of both mCH and 5hmC. Emerging evidence for a dynamic role for the DNA methylation and demethylation machinery in the cellular response to neural activity and in plasticity raises many intriguing questions. As we have argued, understanding the potential role of DNA methylation in cognitive processes such as learning and memory requires integrating information across scales, from the whole genome to individual gene promoters and even single modified bases, potentially ultimately requiring single cell resolution. It also requires methods for interrogating dynamic DNA methylation and determining the kinetics of such processes. To date, such studies have been limited by the requirement for relatively large quantities of genomic DNA for genome-scale methylome profiling. Furthermore, how such dynamic DNA methylation may be specifically targeted to highly localized genomic regions in an activity-dependent manner is currently unknown, and will likely require in depth studies into the targeting of the DNA methylation and demethylation machinery by specific proteins or non-coding RNAs that have the capacity to facilitate sequence-specific recruitment.

A key open question is the relative distribution of DNA methylation (mCG, mCH, and 5hmC) across specific neuronal and glial cell populations. Both excitatory and inhibitory neurons respond to neural activity by expressing common early-response transcription factors, such as *Npas4*, yet the downstream consequences are highly cell type-specific (Spiegel et al., 2014). We would thus expect that DNA methylation, in particular dynamic methylation patterns that may be involved in the cellular response to activity, will be distinctly regulated in different excitatory and inhibitory neural cell types. Supporting this, the abundance and distribution of 5hmC is cell type-specific in the cerebellum, where Purkinje neurons, granule cells and Bergmann glia harbor distinct amounts of the mark (Kriaucionis and Heintz, 2009; Mellén et al., 2012). Addressing this issue is challenging because it requires purifying nuclear material from defined cell types. Recent advances in cell sorting using marker genes (Molyneaux et al., 2015), transgenic mouse lines (Sugino et al., 2014), nuclei labeling and isolation (Deal and Henikoff, 2010), as well as single-cell analysis (Smallwood et al., 2014; Farlik et al., 2015) will help to uncover the role that DNA methylation is playing at the level of specific neuron types (Mo et al., 2015). By combining these molecular techniques with behavioral and systems neuroscience approaches, we may soon be positioned to learn whether DNA methylation is a necessary but merely permissive enabler of learning and memory, or whether DNA methylation and other epigenetic regulatory mechanisms are more deeply involved in the information processing function of the brain.

## Conflict of interest statement

The authors declare that the research was conducted in the absence of any commercial or financial relationships that could be construed as a potential conflict of interest.
